# Astaxanthin mitigates radiation-induced erectile dysfunction: protective effects on corpus cavernosum in a rat model

**DOI:** 10.1038/s41443-025-01106-6

**Published:** 2025-06-13

**Authors:** Gunal Ozgur, Bahadir Sahin, Beste Melek Atasoy, Canberk Tomruk, Cansin Sirin Tomruk, Hasan Huseyin Tavukcu, Ali Yaman, Cemile Ceylan, Deniz Mukaddes Turet, Sehkar Oktay, Yigit Uyanikgil, Gonca Haklar, Haydar Kamil Cam

**Affiliations:** 1 Marmara University Pendik Training and Research Hospital, Department of Urology, Istanbul, Turkey; 2 Department of Urology, School of Medicine, Marmara University, Istanbul, Turkey; 3 Department of Radiation Oncology, School of Medicine, Marmara University, Istanbul, Turkey; 4 Department of Histology and Embryology, School of Medicine, Ege University, Izmir, Turkey; 5 Department of Urology, Camlica Hospital, Medipol University, Istanbul, Turkey; 6 Department of Biochemistry, School of Medicine, Marmara University, Istanbul, Turkey; 7 Department of Radiation Oncology, Private Istanbul Oncology Hospital, Istanbul, Turkey; 8 Experimental Animal Implementation and Research Center, School of Medicine, Marmara University, Istanbul, Turkey; 9 Department of Basic Medical Sciences, Faculty of Dentistry, Marmara University, Istanbul, Turkey

**Keywords:** Erectile dysfunction, Experimental models of disease

## Abstract

The objective of this study was to evaluate the effects of ionizing radiation (iR) on corpus cavernosum and the potential of astaxanthin (AST) in preventing radiation-induced erectile dysfunction (RiED). Male Wistar Albino rats (10–12 week, 250–300 g) were divided-into four groups: sham (SH, *n* = 8), radiotherapy (RT, *n* = 8), vehicle-administered (olive oil (OO); RT + OO, *n* = 12), and astaxanthin (RT + AST, *n* = 12). The RT-group received 12-Gy prostate-targeted iR. The vehicle-administered (OO) group received iR with daily 1 ml OO via oral gavage, while the AST-group received iR with 50 mg/kg AST dissolved in OO. After the treatment-period (12-week), intracavernosal pressure to mean arterial pressure (ICP/MAP) ratios in the RT [0.28(0.14–0.65)] and OO groups [0.26(0.19–0.64)] were significantly lower than in the SH [0.6(0.43–0.72)] and AST [0.53(0.35–0.64)] groups (*p* < 0.05). iR caused narrowing of the cavernous sinusoids (RT:95.38 (84.62–110.05) vs SH:132.33 (113.27–155.86), AST:124.44 (112.11–131.97) µm, *p* < 0.001). Alpha smooth muscle actin (SH:165 (136.25–188.75) vs RT:100 (87.5–112.5), AST:137.5 (107.5–155), *p* < 0.001), endothelial nitric-oxide synthase (NOS) (SH:127.5 (115–167.5) vs RT:92.5 (81.25–98.75), AST:115 (86.25–128.75), *p* = 0.002) and neuronal NOS (SH:152.5 (133.75–163.75) vs RT:95 (81.25–103.75), AST:135 (125–140), *p* < 0.001) were diminished in the RT-group and preserved in the AST-group according to immunohistochemical scoring. Biochemical measurements of the corpus cavernosum revealed that the level of cGMP was significantly higher (93.15 (71.22–103.38) vs 70.8 (65–72.35) pmol/ml) in the AST-group, while lipid peroxidation was significantly higher (32.38 (29.07–36.98) vs 20.14 (17.85–21.04) nmol.mda/g) in the RT-group (*p* = 0.004, *p* < 0.001). This trial showed that AST preserved ICP/MAP values and histopathological-biochemical parameters after exposure to iR.

## Introduction

Prostate cancer is the most common cancer among men in developed countries and the risk of development from birth to death is approximately 1 in 8 [1]. Radiotherapy (RT) is a widely utilized curative treatment option for localized prostate cancer as an alternative to radical surgery [2, 3]. However, detrimental effects of RT on healthy surrounding tissues are inevitably major concern for the patients. It is known that the toxicity of RT to bladder, intestinal and penis tissue causes various complications [4, 5]. Among them, penile tissue damage can lead erectile dysfunction (ED). Approximately 40% of patients develop ED after 3 to 5 years following RT [6].

In rat models, a time-dependent decrease in intracavernosal pressure (ICP) was reported, and oxidative stress was postulated to be a causative mechanism for radiation-induced erectile dysfunction (RiED) [7, 8]. Therefore, it is thought that reducing oxidative stress may prevent the development of RiED. Consequently, a variety of antioxidants were searched to preclude negative effects of RT [9–12]. Astaxanthin (AST) (3,3′-dihydroxy-β-β‘-carotene-4,4′-dione) is a strong antioxidant carotenoid commonly found in seafood. AST has beneficial effects in reducing inflammation, cardiovascular diseases, perfusion-reperfusion injury and oxidative damage [13]. A protective role of AST for raditon-induced lung injury was observed in a rat model [14]. Although AST, a potent antioxidant, has been studied in various studies in both urologic (kidney, prostate, testis) and other organs (cardiovascular, liver, lung), there is no experimental model searching the role of AST for RiED [13–17].

The aim of this study was to evaluate the possible protective effects of AST supplementation on the prevention of ED by measuring the immunohistochemical and biochemical parameters in a rat model of pelvic RT.

## Materials and methods

### Animals and design of experiment

The experimental study was approved by the Institutional Animal Care Ethics Committee (No:27.2021.mar). The rats were fed ad libitum at 21 °C temperature, 12 h dark and 12 h light cycles. A total of 40 Wistar Albino rats (male, 10–12 weeks old, 250–300 g) were randomly divided into four groups: sham (SH), radiotherapy (RT), vehicle-administered (olive oil (OO); RT + OO), and astaxanthin (RT + AST). All rats were anesthetized with 10 mg/kg xylazine and 100 mg/kg ketamine. The first SH group (*n* = 8) received only anesthesia. The RT group (*n* = 8) had 12-Gy prostate-targeted iR using volumetric modulated arc therapy method [18]. OO group (*n* = 12) had iR + 1 ml OO. The forth AST group (*n* = 12) had iR + 50 mg/kg AST (Astaxanthin, AmBeed, Illinois, USA) dissolved in 1 ml of OO. The iR procedure was performed by a single radiation oncologist using the Elekta Versa HD (Stockholm, Sweden) with agility multi-leaf collimator system.

The treatment (AST) group received the antioxidant agent through oral gavage for 12 weeks, starting one day after iR. AST was used at different doses for different organs such as liver (5 and 100 mg/kg AST), kidney (25 and 75 mg/kg AST) and prostate (40 and 80 mg/kg AST) [15–17]. For this reason, a dose of 50 mg/kg AST, which is higher than the lowest doses shown to be effective in other tissues in previous studies and in the effective dose range, was preferred for the study. At the end of the 12-week experimental period, mean arterial pressure (MAP) and ICP measurements were performed under anesthesia. Then, the rats were sacrificed, and tissues were removed [8, 19]. In this study, the investigators who performed ICP/MAP measurements, histological, biochemical, and statistical analyses were blinded to the experimental groups throughout the procedures.

### Intracavernosal pressure/mean arterial pressure (ICP/MAP) measurement after stimulation of cavernosal nerve

**Fig. 1:**
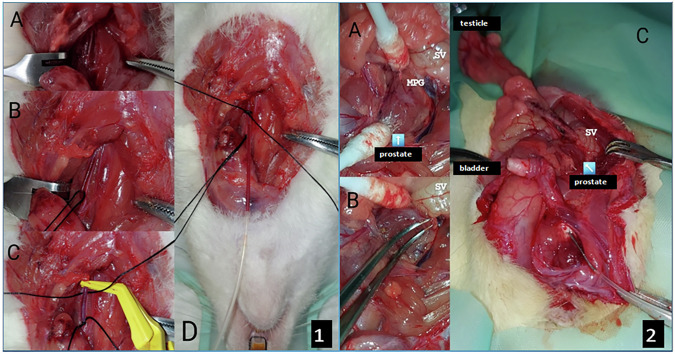
Intracavernosal pressure/mean arterial pressure measurement in the rat. 1. ICA Cannulation: **A** Accessing ICA and vagus nerve by neck dissection. **B,** **C** Isolating ICA by separating it from the vagus nerve. **D** Partially cutting ICA with microscissors and cannulating it with a heparinized (250 U/ml) polyethylene-50 (PE-50) tube (COMBAT Instruments, Ankara, TR). After the bulldog clamp is opened, blood flow pulsation inside the cannula is observed. 2. Isolation of CN and Cannulation of CC: **A** Finding MPG and CN on the lateral side of the prostate. **B** Dissecting CN from surrounding tissues. **C** Placing the cannula into CC. *MPG Major Pelvic Ganglion, *SV Seminal Vesicle, *ICA Internal Carotid Artery, *CN Cavernosal nerve, *CC Corpus Cavernosum.

**Fig. 2:**
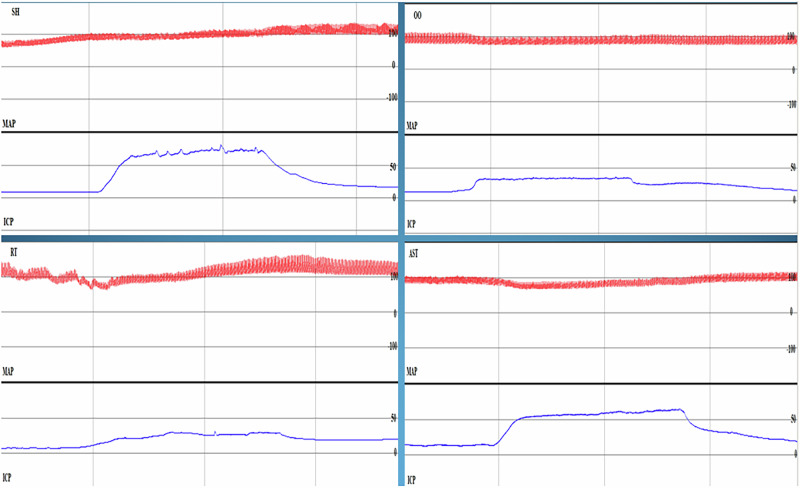
Intracavernosal pressure/mean arterial pressure measurement graphs. ICP/MAP Measurements: An example graph is shown for each group. The upper (red) line represents the arterial pressure (mm-Hg) and the lower line (blue) represents the corpus cavernosum pressure (mm-Hg). Pressure changes after electrical stimulation of the cavernous nerve can be seen. The mean arterial pressure and the highest corpus cavernosum pressure were divided each other and ICP/MAP values were reached. *SH Sham, *RT Radiotherapy, *OO Olive Oil, *AST Astaxantin.

The nerves innervating the rectum, bladder and the cavernosal nerve (CN) exit from the major pelvic ganglion posterolateral to the prostate gland in rats [20]. ICP measurement and simultaneous MAP measurement were performed after electrical stimulation of the CN to evaluate the erectile function of rats [21, 22]. Under anesthesia, the internal carotid artery was cannulated by neck dissection (Fig. 1A–D). The abdomen was opened with a midline incision, the bladder was retracted to the right lateral side, the left testicle was removed and retracted superiorly and to the right lateral side, and the prostate was exposed (Fig. 2A). Major pelvic ganglion and CN were dissected from the surrounding tissues (Fig. 2B). The penile skin was degloved and the corpus cavernosum (CC) was reached by exploration of the bulbocavernosus muscles and entered with a 24-gauge needle (Fig. 2C). CN was stimulated just distal to the major pelvic ganglion with a bipolar electrode with 1.5 mA, 20 Hz, pulse width 5 ms, 35 ms delay, at 7.5 V parameters. ICP and MAP measurements were recorded with a MP-35 data-acquisition system (COMMAT Instruments, Ankara, TR) utilizing a Biopac Student Lab PRO recording software (Biopac Systems Inc., CA, USA). ICP/MAP ratio was calculated as the highest ICP value recorded during stimulation divided by the mean MAP value [22, 23].

### Histological analysis

After ICP/MAP measurement, the rats were sacrificed and penile tissue was removed. This penile tissue including CC and corpus spongiosum was divided into two parts and one part was preserved in 4% formaldehyde at + 4C in a light-free environment for histochemical and immunohistochemical examinations. Haematoxylin-Eosin staining, Masson Trichrome staining and immunohistochemical staining with endothelial nitric oxide synthase (eNOS) (Novus Biologicals, NB300-500, UK), neuronal nitric oxide synthase (nNOS) (BIOSS, BS-0156R, USA), alpha-smooth muscle actin (α-SMA) (Novus Biologicals, NBP1-30894, UK) primary antibodies were performed. Cavernous sinusoid widths were measured at ten different points on each tissue specimen using ImageJ (ImageJ software, v1.54 f, National Institutes of Health, Bethesda, Maryland, USA) program. Fibrosis intensity and immunoexpression intensity in immunohistochemical stainings were classified on a scale from 0 to 3 positivity and a H-score was calculated for statistical analysis.

### Biochemical analysis

The other piece of CC was kept at −80 C for biochemical evaluation. Tissues were homogenized and measured as described in commercial ELISA kits. Total Antioxidant Status (TAS) and Total Oxidant Status (TOS) were measured with a Rat TAS (TAS-BT-LAB, E4350, China) and a Rat TOS (TOS- BT-LAB, E1599, China) ELISA kits. Rat superoxide dismutase (SOD) ELISA kit (AFG SCIENCE, EK720188, Northbrook, USA), Rat glutathione (GSH) ELISA kit (AFG SCIENCE, EK720391, Northbrook, USA) and Rat cyclic guanosine monophosphate (cGMP) ELISA kit (AFG BIOSCIENCE, EK720601, Northbrook, USA) were used for the measurement of SOD protein amount, glutathione GSH level and cGMP level. Biochemical methods specified in the literature were used for SOD Activity [24] and lipid peroxidation (LPO) [25]. TECAN Infinite 200 device (Switzerland) was used for absorbance measurements.

### Statistical analysis

Statistical analyses were evaluated using the IBM SPSS Statistics Version 22 package program. The normality of the variables was analyzed visually (histograms and probability graphs) and analytically (Kolmogorov-Smirnov/Shapiro-Wilk tests) methods. The data were statistically analyzed using the Kruskal-Wallis test method for more than two independent groups for numerical (no normal distribution) and ordinal data. The *p*-value < 0.05 was accepted as statistically significant in the analysis of the data. In addition, Bonferroni correction was made for the *p*-value when the groups were compared post-hoc pairwise with Man-Whitney U test. G Power program was used for power analysis. Power analysis was performed on the number of groups and measurement values obtained in previous studies on RiED [19, 23]. We calculated a sample size of at least 8 rats per group to obtain an alpha error level of 0.05 and a power of 0.80 for ICP/MAP measurements. Cliff’s delta was calculated as a non-parametric measure of effect size to quantify the magnitude and clinical relevance of pairwise differences. Cliff’s delta values were interpreted based on the absolute value as negligible (<0.11), small (0.11–0.27), medium (0.28–0.42), and large (≥0.43).

## Results

Power analysis revealed at least 8 rats were required for each group. The OO and AST groups were designed to have addional rat in considering the possible animal loss during daily procedure. In the end of study, one rat died each from OO and AST groups. Finally, the results obtained from 8 rats in each SH and RT groups and 11 rats in each OO and AST groups were included in the study.

### Intracavernosal pressure/mean arterial pressure (ICP/MAP) measurements

An example of ICP/MAP measurement graphs from each group is shown in Fig. 2. The median (minimum-maximum) values (mm-Hg) of ICP/MAP measurements were 0.6 (0.43–0.72) for the SH group, 0.28 (0.14–0.65) for the RT group, 0.26 (0.19–0.64) for the OO group and 0.53 (0.35–0.64) for the AST group. It was found that the RT group and the OO group had lower ICP/MAP measurements than the SH group at a statistically significant level (*p* = 0.009, *p* = 0.002 respectively). In addition, the AST group exhibited higher ICP/MAP measurements compared to the RT and OO groups (*p* = 0.01, *p* = 0.03 respectively).

### Histochemical and immunohistochemical findings of corpus cavernosum tissues

**Fig. 3:**
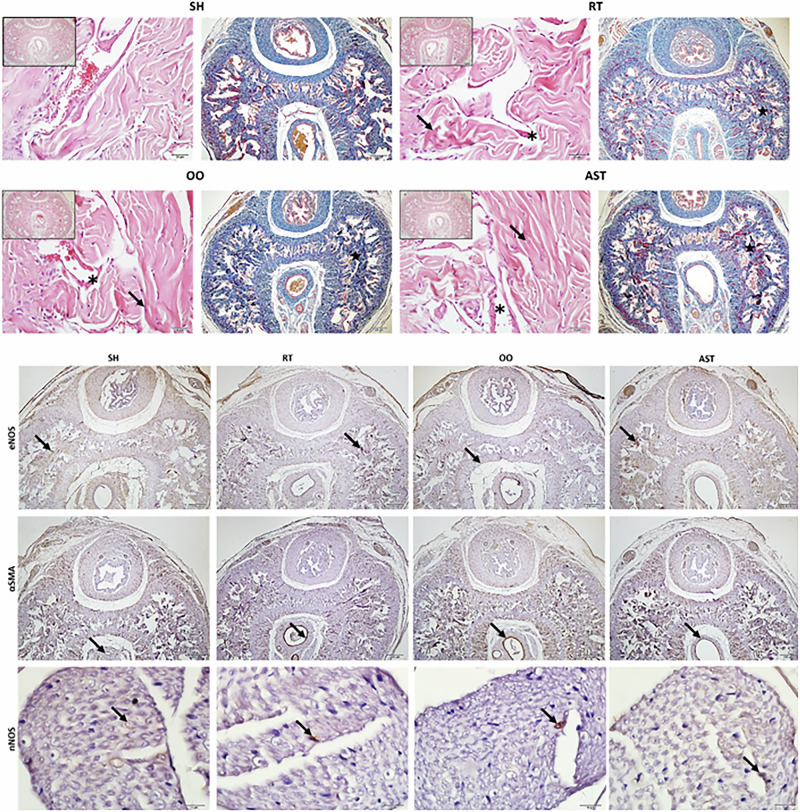
Histochemical and immunohistochemical findings of corpus cavernosum tissue. 1. H&E(x40) and Masson Trichrome(x4) staining: SH: Normal histology, RT: Disorganised collagen bundles and fibrotic changes (arrow), narrowing of the cavernous sinuses (star) and endothelial damage (asterisk) were observed, AST: Minimal endothelial damage (asterisk), normal-appearing cavernous sinuses (star), more regular collagen bundles (arrow) and less fibrotic areas were observed. 2. eNOS (x4 magnification), αSMA (x4 magnification) and nNOS (x40 magnification) immunoexpression: intensities of eNOS, αSMA and nNOS tended to decrease in RT and OO groups compared to SH group; these changes were less in AST group. Arrow: Positive immunoexpression. *eNOS Epithelial Nitric Oxide Synthetase, *nNOS Neuronal Nitric Oxide Synthetase, *SMA protein Smooth Muscle Actin Protein, *SH Sham, *RT Radiotherapy, *OO Olive Oil, *AST Astaxantin.

In the SH group; CC, corpus spongiosum and connective tissues had normal histological appearance. In the RT group, collagen fibril bundles in the tunica albuginea were coarse and scattered. Narrowing of the cavernous sinuses (RT: 95.38 (84.62-110-05) µm vs SH: 132.33 (113.27–155.86) µm and AST: 124.44 (112.11–131.97) µm, *p* < 0.001, *p* = 0.007) due to fibrotic areas (SH = 2.5 (0–10) vs RT = 35 (10–55) according to scoring, *p* < 0.001) in the cavernous tissue, damage and oedema in the endothelium lining the cavernous bodies were observed. The OO group had similar findings to the RT group. Collagen bundles were more regular in the AST group compared to the RT and OO groups. Also, fibrotic changes in the CC were less than in the other groups receiving radiotherapy (AST = 15 (5–25) vs RT = 35 (10–55) and OO = 32.5 (10–60) according to scoring, *p* = 0.007, *p* = 0.009) and the endothelial cells lining the cavernous bodies were preserved and underwent mild oedematous change in the AST group (Fig. 3).

**Fig. 4:**
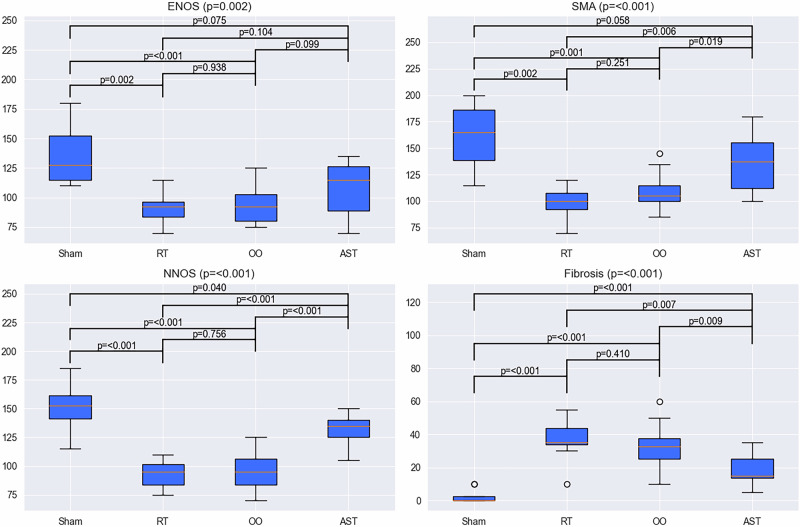
Histochemical and immunohistochemical analysis of corpus cavernosum tissues. The statistical analysis using the H score revealed a significant decrease in the immunoexpression intensities of eNOS, α-SMA, and nNOS (*p* = 0.002, *p* = 0.002, *p* = 0.001) and an increase in fibrosis, in the RT and OO groups compared to the SH group (*p* < 0.001). In the AST group, α-SMA, nNOS immunoexpressions were increased and fibrosis was decreased compared to RT (*p* = 0.006, *p* < 0.001, *p* = 0.007 respectively). Kruskal-Wallis test method was used for more than two independent groups. Man-Whitney U test was used for post-hoc pairwise comparation. *eNOS Epithelial Nitric Oxide Synthetase, *nNOS Neuronal Nitric Oxide Synthetase, *SMA protein Smooth Muscle Actin Protein, *RT Radiotherapy, *OO Olive Oil, *AST Astaxantin.

The median (IQR) values of H-score for SH, RT, OO, and AST groups, respectively, were 127.5 (115–167.5), 92.5 (81.25–98.75), 92.5 (80–107.5), 115 (86.25–128.75) for eNOS; 165 (136.25–188.75), 100 (87.5–112.5), 105 (100–115), 137.5 (107.5–155) for α-SMA; 152.5 (133.75–163.75), 95 (81.25–103.75), 95 (81.25–108.75), 135 (125–140) for nNOS. The statistical analysis of fibrosis and immunexpressions of the markers are shown in the Fig. 4 with box-plot graphs. The statistical analysis conducted using the scores revealed a significant decrease in the immunoexpression intensities of eNOS, α-SMA, and nNOS, as well as an increase in fibrosis, in the RT and OO groups compared to the SH group (*p* = 0.002 and *p* < 0.001, *p* = 0.002 and *p* = 0.01, *p* < 0.001 and *p* < 0.001 respectively). In the AST group, α-SMA, nNOS immunoexpressions significantly increased and fibrosis significantly decreased compared to RT group (*p* = 0.006, *p* < 0.001, *p* = 0.007 respectively). Although eNOS levels increased in AST group, no statistically significant difference was observed between RT and OO groups (*p* = 0.104).

### Biochemical analysis findings of corpus cavernosum tissues

Our biochemical measurements were performed by homogenizing CC tissue, and the results are shown in Table 1. cGMP levels were statistically significantly higher in the AST group (*p* = 0.004). Additionally, increased LPO levels were observed in the RT group, significantly higher than the other groups (*p* < 0.001), with lower levels observed in the SH and AST groups compared to the OO and RT groups.

**Table 1:** Biochemical analysis of corpus cavernosum tissues.

Biochemical Markers	GROUPS / Median (IQR)				
	Sham Group (SH)	Radiotherapy Group (RT)	Olive Oil Group (OO)	Astaxanthin Group (AST)	p*
TAS (U/ml)	13.5 (11.9–15.6)	12.8 (10.5–13.65)	9.7 (9.25–11.85)	14.5 (13.32–15.42)	0.054
TOS (U/ml)	8.0 (5.55–8.82)	9.5 (6.3–11.95)	8.3 (6.75–10.45)	7.7 (4.9–10.48)	0.727
cGMP (pmol/ml)	58.0 (54.55–73.85)	70.8 (65.03–72.35)	83.6 (76.65–85.4)	93.15 (71.22–103.38)	**0.004**
GSH (ng/l)	1373.8 (1251.05–1564.3)	1306.6 (1178.85–1454.48)	1527.1 (1259.8–1687.9)	1605.9 (1500.12–1662.62)	0.113
SOD (pg/ml)	3897.45 (3626.35–3975.65)	3827.25 (3621.7–4908.45)	5037 (4199.95–5389.75)	4728.25 (4177.9–5390.5)	0.121
SOD (U/g)	7.29 (6.62–7.3)	5.4 (4.32–5.54)	7.82 (4.32–7.02)	6.75 (5.26–8.1)	0.115
LPO (nmol.mda/g)	18.36 (17.08–20.53)	32.38 (29.07–36.98)	20.91 (19.38–22.7)	20.14 (17.85–21.04)	**0.000**

*TAS* total antioxidant status, *TOS* total oxidant status, *cGMP* cyclic guanosine monophosphate, *GSH* glutathione, *SOD* superoxide dismutase, *LPO* lipid peroxidation.

*Kruskal-Wallis test method was used. Bold values indicate statistically significant differences (*p* < 0.05).

In our study, although TOS levels were slightly elevated in the RT group and TAS levels higher in the AST group, overall statistical significance was not reached (*p* = 0.727, *p* = 0.054, respectively). However, pairwise effect size analysis (Cliff’s delta) showed that the TAS levels in the AST group were significantly higher compared to OO (delta = −0.61, *p* = 0.015), suggesting meaningful antioxidant improvement in AST treatment.

Although GSH levels were higher in the AST group compared to RT, the overall difference was not statistically significant (*p* = 0.113). Nevertheless, pairwise comparisons revealed moderate effects, indicating meaningful higher GSH levels in AST versus both RT and SH groups (delta = −0.56, *p* = 0.039), highlighting potential biological significance despite the marginal statistical results.

For SOD, the amount and activity were separately assessed. Although SOD protein levels appeared lower in the SH group than in other groups, and SOD activity was notably decreased in the RT group while increased in SH and AST groups, there was no statistical significance after Bonferroni correction (*p* = 0.121 for protein, *p* = 0.115 for activity). Pairwise comparisons confirmed moderate effects for protein amount (SH vs. AST, delta = −0.54, *p* = 0.047) and activity (RT vs. AST, delta = −0.54, *p* = 0.048), suggesting biologically relevant differences despite not meeting adjusted statistical significance. This may reflect limited statistical power due to our sample size.

## Discussion

Radiotherapy is effectively used in prostate cancer treatment. After RT, the risk of ED progressively increases in patients. It is thought that the oxidative stress caused by RT plays a role in the development of RiED by causing smooth muscle cell loss, fibrosis, and endothelial cell damage [8, 26]. Prevention of tissue damage by reducing oxidative stress in the treatment of RiED may reduce the development of RiED. AST is a very potent antioxidant molecule that has been used in numerous studies for its efficacy in diabetes, metabolic syndrome, cardiovascular diseases, chronic inflammatory diseases, gastrointestinal diseases, liver diseases, eye diseases, skin diseases, male infertility, and renal insufficiency [13]. There is also the advantage that the availability of currently used oral supplement forms of AST provides accessibility. The present study is the first experimental model to investigate the role of AST for RiED.

ICP/MAP measurement is a known test used to evaluate the erectile function of rats [23]. It was found that ICP/MAP measurements decreased progressively at 2, 4 and 9 weeks after 20 Gy iR therapy in rats [7]. Another experimental study reported that ICP/MAP measurements decreased in the groups administered 15 Gy, 20 Gy and 25 Gy iR and the decrease of ICP/MAP measurements was correlated with iR dose in a 9-week period [19]. Lee et all demonstared that pelvic iR can also cause ED at a dose of 12.5 Gy in rats [27]. In our study, the follow-up period of the rats was longer (12-week) than the literature and we found that ICP/MAP measurements in the RT group after 12 Gy iR were lower than the SH group. A similar study also showed that IC/MAP measurements were lower in rats receiving iR at the 10th week compared to the control group, and resveretrol, an antioxidant molecule, increased ICP/MAP measurements (ICP/MAP ratios: 0.48 vs 0.24, *p* < 0.001) [23]. Liu at all postulated that improvement in ICP/MAP measurements (ICP/MAP ratios: 0.59 vs 0.43, *p* < 0.01) was found in the group given YS-10 supplementation in the RiED model [28]. When the effect of antioxidant molecules on ICP/MAP measurements was compared to RT groups, a 27% amelioration was found in AST group compared to 16% in YS-10 and 24% in resveretrol [23, 28]. The current trial confirmed that AST as an antioxidant, improved erectile functions based on the ICP/MAP measurements in iR rat model.

Previous studies showed that development of fibrosis in penile tissue, loss of α-SMA expression and alteration of eNOS levels in CC observed after iR in rats [8, 26]. There are various studies on the prevention of these changes by antioxidant molecules. Arginine and glutamine supplementation has a decreasing effect on iR-induced changes in the CC in a rat model [9]. Neuronal nitric oxide (nNO) and endothelial nitric oxide (eNO) are important mediators of penile erection [29]. NO increases cGMP production via guanylate cyclase. nNOS expression decreased in rats receiving iR in the long-term and these values improved with resveratrol treatment [23]. In our study, fibrotic areas in CC, narrowing of the cavernous sinusoids due to fibrosis and damage to the endothelial cells lining the cavernous were observed in the RT group. In addition, immunohistochemical evaluation showed that eNOS, nNOS and α-SMA immunoexpression decreased in the RT group. These changes were preserved with the AST treatment.

Patients with ED have increased plasma reactive oxygen radicals and decreased antioxidant levels [30]. Similarly, serum TOS levels are measured high and TAS levels are measured low in patients with ED [31]. Similarly, animal studies have investigated the possibility of similar changes at the tissue level. It has been shown that free oxygen radicals and inflammation markers increase in CC tissue due to oxidative stress [7]. It was observed that GSH level which is an antioxidant molecule, decreased in the CC tissue in the RiED model in rats and these levels increased with antioxidant resveratrol treatment [23]. Although there was no statistically significant alteration in our study, TOS level was slightly higher in RT group, TAS level was higher in AST group, GSH level was slightly lower in RT group and slightly higher in AST group.

cGMP is a very important mediator involved in the formation of erection by relaxing smooth muscles. It is known to be a contributing factor in the pathogenesis of various causes of ED [19]. In the RiED model, it was observed that cGMP levels decreased after RT and cGMP levels improved in the group given antioxidant resveretrol [23]. In our study, there was no statistically significant difference in cGMP levels between the SH group and the RT group, but cGMP levels in the AST group were statistically significantly higher than the SH and RT groups.

SOD is an antioxidant molecule and SOD activity reflects the antioxidant capacity of tissues. SOD activity decreases in diabetes-induced ED model in rats [32]. It was observed that SOD activity decreased in the CC tissue of rats receiving RT in long-term follow-up and improved with resveratrol treatment [23]. SOD protein undergoes various regulation processes after it is produced, as well as required to complex with various elements such as copper and zinc [33]. Some deficiencies of SOD protein like post-translational modifications and incorporation with ions, affect the SOD activity of the tissue. Therefore, a decrease in antioxidant capacity would be expected in the lack of maturation of SOD protein. An increase in SOD protein amounts and a decrease in SOD activity were observed due to oxidative stress in acute and chronic lung injury in a study [34]. Although no statistically significant change observed in our measurements, it was observed that the amount of SOD protein in the tissue increased in the RT groups, but SOD activity decreased in the RT group and increased in the astaxanthin-treated group. It may be interpreted that the organism works to increase the amount of SOD protein at the tissue level due to the cellular stress in the RT-treated groups, but oxidative stress and cell dysfunction may decrease the antioxidant activity of SOD protein.

Free oxygen radicals cause LPO by reacting with the bonds of cholesterol and unsaturated fatty acids in the cell membrane. LPO in cell membranes leads to cellular damage [35]. iR also rises LPO in cells [36]. So, LPO increases in tissues after iR [7]. Antioxidant molecules are known to be radioprotective by reducing the stress in cells [37]. We found lower LPO levels in the AST group.

In summary, this experimental model provides new insights in concept of RiED. First of all, we observed that a 12 Gy dose of iR caused a significant reduction in the ICP/MAP ratio in rats. AST exhibited an improvement in these ICP/MAP values. Besides histopathological analysis revealed increased fibrotic deposition and a concomitant reduction in the diameter of cavernous sinusoids, as well as endothelial cell damage in CC tissues after iR. Additionally, a decline in the immunoexpression of eNOS, nNOS, α-SMA was noted in the RT group. Biochemically, iR was observed to alter tissue levels of oxidative and antioxidative agents. Notably, the AST treatment group displayed a statistically significant elevation in cGMP, an essential mediator in the physiology of erection, and a significant reduction in LPO levels, an indicator of oxidative stress, compared to the RT group. These findings suggest that AST, with its antioxidative capacity, could potentially counteract the histological and biochemical alterations induced by iR in rat CC tissue.

There are some limitations in our study. First of all, this is an animal study and there would be natural limitations in predicting the clinical translation of these study findings. Due to the study design, the adverse effects of iR and the potential preventive effect of AST were evaluated after twelve weeks follow-up. However, we could not examine delayed effects of iR and AST. The absence of a dose-response analysis is an another limitation of our study.

It is clear that pelvic RT progressively causes ED as one of the major complications. The underlying mechanism of RiED involves the generation of oxidative stress. Our 12-week experimental model confirmed that a 12 Gy dose of iR caused a significant reduction in the ICP/MAP ratio in rats. Additionally, significant alterations in histological and biochemical parameters associated with iR were observed. AST, as a potent antioxidant, improved ICP/MAP values and histological and biochemical markers, such as fibrosis, nNOS, α-SMA, cGMP, and LPO. Therefore, the results of this experimental study, for the first time, suggest that AST can prevent the development of RiED and provide a basis for further clinical trials.

## Data Availability

The data that support the findings of this study are available from the corresponding author upon reasonable request.
